# Fast food purchasing and access to fast food restaurants: a multilevel analysis of VicLANES

**DOI:** 10.1186/1479-5868-6-28

**Published:** 2009-05-27

**Authors:** Lukar E Thornton, Rebecca J Bentley, Anne M Kavanagh

**Affiliations:** 1 Key Centre for Women's Health in Society, Melbourne School of Population Health, The University of Melbourne, Victoria, 3010, Australia

## Abstract

**Background:**

While previous research on fast food access and purchasing has not found evidence of an association, these studies have had methodological problems including aggregation error, lack of specificity between the exposures and outcomes, and lack of adjustment for potential confounding. In this paper we attempt to address these methodological problems using data from the Victorian Lifestyle and Neighbourhood Environments Study (VicLANES) – a cross-sectional multilevel study conducted within metropolitan Melbourne, Australia in 2003.

**Methods:**

The VicLANES data used in this analysis included 2547 participants from 49 census collector districts in metropolitan Melbourne, Australia. The outcome of interest was the total frequency of fast food purchased for consumption at home within the previous month (never, monthly and weekly) from five major fast food chains (Red Rooster, McDonalds, Kentucky Fried Chicken, Hungry Jacks and Pizza Hut). Three measures of fast food access were created: density and variety, defined as the number of fast food restaurants and the number of different fast food chains within 3 kilometres of road network distance respectively, and proximity defined as the road network distance to the closest fast food restaurant.

Multilevel multinomial models were used to estimate the associations between fast food restaurant access and purchasing with never purchased as the reference category. Models were adjusted for confounders including determinants of demand (attitudes and tastes that influence food purchasing decisions) as well as individual and area socio-economic characteristics.

**Results:**

Purchasing fast food on a monthly basis was related to the variety of fast food restaurants (odds ratio 1.13; 95% confidence interval 1.02 – 1.25) after adjusting for individual and area characteristics. Density and proximity were not found to be significant predictors of fast food purchasing after adjustment for individual socio-economic predictors.

**Conclusion:**

Although we found an independent association between fast food purchasing and access to a wider variety of fast food restaurant, density and proximity were not significant predictors. The methods used in our study are an advance on previous analyses.

## Background

Fast food consumption has been associated with increased risk of adverse health outcomes including increased body weight [1,2] and diabetes [1]. Evidence from the US showed that recent increases in the rates of fast food consumption [3,4] have coincided with growth in the number of fast food restaurants [5].

Despite increases in both consumption and availability of fast food the potential link between the two remains largely unexplored with only Jeffery and colleagues in the U.S. [6] and Turrell and Giskes [7] in Australia undertaking detailed investigation on this. These studies found no statistically significant link between greater access to fast food and increased fast food purchasing. Unfortunately, these studies have several methodological problems. First, Turrell and Giskes [7] assessed density and proximity using access measures created from the centroid of a study area which leads to 'aggregation error' [8] because the area-level variable may not be an accurate measure of individual exposure. Second, both studies did not ask questions about the consumption or purchasing of particular fast food brands but defined the exposure variable with more specificity, resulting in a lack of congruence between exposure and outcome variables. For example, although Turrell and Giskes created specific categories of takeaway stores for the exposure variable, the outcome variable related to purchasing was asked as a general question and could not be specifically matched to any of the exposure categories. Third, studies of access to fast food restaurant and purchasing are hampered by confounders [9,10] because they failed to account for the fact that the association between fast food restaurant accessibility and purchasing may be a function of *demand *for fast food driving supply rather than *supply *(or accessibility) influencing demand (i.e. endogeneity). For example, Subramanian *et al*. have argued that fast food chains may open in areas because of the taste preferences of local residents [9] and previous research has supported an association between taste preferences and fast food consumption among adolescents [11]. Prior research has also indicated that food preferences and other attitudes such as convenience and health may influence food choices [12,13]. Therefore, to extend Subramanian *et al*.'s argument, it is also plausible that the attitudes of local residents may also influence the location of fast food restaurants. In addition, a key to the success of major fast food chains has been their ability to target populations based on demographic and socio-economic criteria [14]. Isolating an independent association between fast food access and purchasing requires methods that account for these potential confounders.

The primary aim of this analysis was to determine if better access to fast food restaurants in the local neighbourhood environment was a significant predictor of fast food purchased for consumption at home. We improved on previous research by addressing the issues of aggregation error, lack of specificity of exposures and outcomes and confounders in a multilevel study of 2547 participants in 49 areas in metropolitan Melbourne, Australia. We tested the associations between access to five major fast food chains and purchasing fast food from these same chains, using three measures of accessibility to fast food restaurants (density, variety, and proximity) measured from participants' homes. The potential magnitude of these effects was estimated before and after adjustment for potential confounders.

## Methods

We analysed data collected between September and December 2003 as part of the Victorian Lifestyle and Neighbourhoods Environment Study (VicLANES). Information on the data collection and analytical methods are outlined below while further details related to the VicLANES data have been published elsewhere [15-17]. The VicLANES project design was approved by the La Trobe University Human Ethics Committee.

### Study area and population

Data collection for VicLANES was undertaken within metropolitan Melbourne extending approximately 30 kilometres from the Central Business District (CBD). All Census Collector Districts (CCDs) (the size of a CCD is approximately 225 dwellings) within the 21 innermost Local Government Areas (LGAs) of the Melbourne metropolitan area were ranked and stratified into septiles according to the proportion of households with a weekly pre-tax income of less than $400 per week (a measure of disadvantage). Fifty CCDs were then randomly chosen for sampling from the least disadvantaged (n = 17), mid disadvantaged (n = 16) and most disadvantaged (n = 17) septiles. A Food Purchasing survey that asked extensive information about food purchasing patterns, including fast food, was mailed to a total of 3995 households who were randomly selected from the electoral roll (voting is compulsory for Australian Citizens) within the selected CCDs. This survey was completed by the person who undertook the majority of food shopping for that household. A total of 2564 valid responses to this survey were received (64% response rate).

### Fast food purchasing

The outcome of interest was fast food purchased from five major fast food chains (Red Rooster, McDonalds, Kentucky Fried Chicken, Hungry Jacks, and Pizza Hut) for consumption at home. Participants were asked to answer how often fast food was consumed at home from each of these fast food chains over the last month with six response categories listed (not at all, one time, 2–3 times, 4–6 times, 7–10 times, or 11+ times). The total amount purchased from each of the five restaurants was summed together and then categorised (midpoint values used for sums e.g. 2–3 times category scored as 2.5; 11 used for 11+ times category). By asking what had been eaten at home it was assumed that the product had been bought close to home so as to still be warm upon consumption. The resulting dependent variable was defined as those who have not eaten fast food over the past month (never), those who have eaten it a total of one to three times over the last month (monthly), and those who have eaten it a total of four or more times over the last month (weekly). In effect these categories represented those that never consume fast food at home, and those that consume fast food at home either infrequently or frequently.

### Accessibility to fast food restaurants

All fast food restaurants from the five major chains of interest were geocoded across metropolitan Melbourne using address information from the 2003/04 Melbourne White Pages phone directory.

Three measures of access were created: density, variety, and proximity. Density was defined as the total number of fast food restaurants within 3 kilometres of road network distance from each participant's household location. Variety was created using the same approach but instead a count was made of the number of different fast food restaurant chains within 3 kilometres of a person's residence. Proximity was defined as the road network distance to the nearest fast food restaurant from each participant's household. For density and variety a 3 kilometre road network distance was chosen in accordance with previous research that has shown that most people do their food shopping within 3.2 kilometres of their home [18] and has since been used as a measure of driving distance to stores [19].

The Network Analyst extension within ArcGIS 9.2 [20] was used to undertake the network analysis. The spatial datasets for the road (Vicmap Transport) and address (Vicmap Address) information were obtained through the Victorian Department of Sustainability and Environment. One-way restrictions were accounted for in analysis.

### Confounders

#### Individual and household variables

Data on demographic and socio-economic variables we conceptualised as confounders were obtained from VicLANES survey responses. Variables related to age, country of birth, education, occupation and attitudes were based on the main food shopper whereas household composition and income were household level variables.

Age was coded into six categories (18–24, 25–34, 35–44, 45–54, 55–64, or ≥ 65 years). Country of birth was a binary variable defined by whether the respondent was born in Australia or overseas. Five categories of household composition were used (single male adult without children, single female adult without children, a single adult with a child or children, two or more adults without children, or two or more adults with a child or children). Sex was defined only for single person households as it was believed that these households may differ in purchasing patterns but households with two or more adults would not be affected by the sex of the main food shopper because in a high majority of case this was a woman. Four categories of education (bachelor degree or higher, a diploma (associate or undergraduate), vocational, or no post school qualification) were created. Respondent occupation was coded to the Australian Bureau of Statistics (ABS) Australian Standard Classification of Occupations (ASCO)) [21] with the final categories being professional employees (managers, administrators, professionals, and para-professionals), white-collar employees (clerks, salespersons, and personal service workers), blue-collar employees (tradespersons, machine operator, drivers, labourers, and related workers), or not in the labour force (retired, studying, unemployed, not looking for work, or unable to work). Total household income was coded into five categories using cut points of $20800, $36399, $52000, and $78000. Household level income variables have previously been reported as the best measures for determining relationships with purchasing [22]. Variables for attitude and perception were derived from responses to statements: 1) healthy foods are difficult or time consuming to prepare; 2) most healthy foods aren't very tasty; 3) when buying food for my household, health or body-weight considerations influence my choice. These were coded to binary variables (agree or do not agree).

#### Area-level variables

Area socio-economic disadvantage was classified by the proportion of households within a CCD with a weekly pre-tax income of less than $400 per week. These proportions were categorised with areas defined as least disadvantaged (mean 7.0%, range 3.5% – 8.5%), mid disadvantaged (mean 15.3%, range 14.4% – 16.7%), or most disadvantaged (mean 31.4%, range 24.1% – 59.6%).

### Missing data imputation

Data were missing on a number of key variables including income (35% missing). In total, the proportion of the sample with completely observed data for all variables examined was 61%. Rather than analysing the complete cases only, potentially biasing estimates, missing data was imputed under a Missing At Random (MAR) assumption. Under this assumption the presence of missing values are modelled as a function of observed variables. Ten datasets with imputed values for missing items on each variable were estimated using the user-written command Imputation by Chained Equations (ICE) (P Royston) in Stata 10.1[23]. This uses the conditional densities of variables, given all other variables, to estimate missing values. Multiple imputation is a more rigorous approach than single imputation as it is less biased and more precise [24].

### Descriptive analysis

The different distribution of the proportion of respondents in each of the fast food purchasing categories across the predictor variables were compared using chi-square statistics.

### Multilevel analysis

We undertook final analysis on 49 out of the 50 original VicLANES CCDs (2,547 participants). This was because one CCD had the Melbourne central business district (CBD) within its 3 kilometre network buffer. When examined from its centroid (individual location, not centroids, are used in the main analysis), this CCD had 29 fast food restaurants within 3 kilometre whereas the next highest CCD had nine.

Multilevel multinomial regression was undertaken in Stata 10.1 using the GLLAMM function prefixed by the user-written "mim" command (created by JC Galati, P Royston, and JB Carlin) which allowed for analysis to be undertaken across multiple datasets. Results from the multilevel analysis were presented as odds ratios (OR) with 95% confidence intervals (95% CI) which estimate the odds of fast food purchasing either monthly or weekly compared to the baseline category which was never purchased during last month. For both density and variety the odds relate to an increase of one extra restaurant or chain whereas the odds for proximity relate to an increase in distance of 1 kilometre.

### Conceptual model

In order to carefully consider confounders and mediators of fast food access and purchasing, and hence reduce the possibility of biased estimates, we used Directed Acyclic Graphs (DAGs) [25-27]. DAGs are used to visually represent the assumed causal relationship among exposures, outcomes, and covariates [9,28] and are increasing being used to correctly identify potential confounders and mediators [29-32].

**Figure 1:**
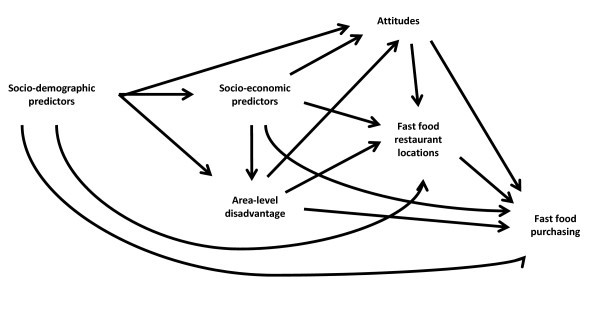
**DAG representing causal relationship between fast food restaurant access and fast food purchasing**.

Figure 1 represents the DAG for these analyses. In this DAG, variables for socio-demographic and socio-economic characteristics, area-level disadvantage, and attitudes are all conceptualised as potential determinants of fast food purchasing and the location of fast food restaurants and thus are potential confounders. Each model builds on the previous to determine where the main attenuation of effects occurs. The ordering of the variables into the models ensures that the effects being modelled are less likely to be biased by confounding. For example, demographic characteristics are likely to influence socio-economic position which in turn is likely to influence area of residence. Each of these factors potentially influence food related attitudes and preferences.

Based on this DAG we ran five models for each measure of access:

**Model 1**: Unadjusted

**Model 2**: Socio-demographic predictors (Age, country of birth and household composition)

**Model 3**: Model 2 + socio-economic predictors (education, occupation and income)

**Model 4**: Model 3 + area-level disadvantage

**Model 5**: Model 4 + food attitudes (related to time pressures and health considerations) and preferences (taste)

The first model run was unadjusted for any confounders. Analysis prior to and after adjustment for confounders allows for the presentation of the upper and lower bound of the effects; within which the "true" effect lies [33]. In Models Two and Three individual and household demographic and socio-economic predictors were added, respectively. These are likely predictors of the decision to purchase fast food and fast food companies understand the demographics of their frequent consumers and are more likely to locate in areas where these characteristics are most common [14]. Model 4 includes area disadvantage as fast food companies may choose to locate in areas based on the summary socio-economic characteristics and these locations are also likely to have cheaper land prices and may be closer to highways and major roads leading to a greater chance of success should the fast food store locate their. Further determinants of store location may exist in the form of attitude variables and taste preferences [9,10]. Kawachi and Subramanian have reported that epidemiological studies have ignored these endogenous predictors and for public health research to move forward such predictors must be acknowledged [10]. The final model includes the variables that capture attitudes and taste.

## Results

### Descriptive statistics

The distribution of fast food purchasing frequency across each of the confounder variables are presented in Table 1. A sample of 2,547 respondents from 49 CCDs were analysed of which 56% never purchase fast food over the last month, 35% purchased on an infrequent basis (1–3 times per month), and 10% on a frequent, weekly, basis (4 or more times per month). More frequent fast food purchasing was highest in younger age groups, households with children, less education, blue-collar employees, lower total household income and living in areas with greater levels of disadvantage. Those who agreed healthy foods were time consuming to prepare or weren't tasty were most likely to purchase fast food on a weekly basis. Fast food was purchased less often when health or body-weight consideration were said to influence food choice. Country of birth was unrelated to fast food purchasing frequency.

**Table 1:** Descriptive data for confounders by frequency of fast food purchasing

	Never	Monthly	Weekly	P-value*
	%	%	%	
*n*.	1424	878	245	
*Proportion who eat fast food*	55.9	34.5	9.6	
*Age (years)*				
18–24	30.4	48.2	21.4	
25–34	40.7	45.4	13.9	
35–44	41.5	43.8	14.7	
45–54	57.3	32.8	9.9	
55–64	70.4	25.0	4.6	
65 or over	73.5	23.9	2.6	<0.001
*Country of birth*				
Australia	56.3	34.4	9.3	
Overseas	55.0	34.6	10.4	0.627
*Household Composition*				
Single adult male – no children	61.2	28.5	10.3	
Single adult female – no children	73.5	22.4	4.0	
Single – with children	43.1	43.1	13.9	
Two or more adults – no children	65.3	28.7	6.0	
Two or more adults – with children	45.2	41.6	13.3	<0.001
*Education*				
Bachelor degree of higher	62.7	31.4	5.9	
Diploma	56.9	34.3	8.9	
Vocational	50.6	38.3	11.1	
No post school qualifications	52.7	35.3	12.0	<0.001
*Occupation*				
Professional	58.8	33.1	8.1	
White-collar	50.8	38.3	10.9	
Blue-collar	38.9	40.3	20.9	
Not in labour force	58.1	33.1	8.8	<0.001
*Income*				
A$78,000 or more	62.1	32.4	5.5	
$52,000 – $77,999	51.9	37.7	10.4	
$36,400 – $51,999	53.4	36.3	10.4	
$20,800 – $36,399	51.9	35.9	12.1	
$20,799 or less	56.8	31.0	12.2	<0.001
*Area-level disadvantage*				
Least disadvantaged	59.3	33.9	6.8	
Mid disadvantaged	57.0	33.5	9.6	
Most disadvantaged	50.6	36.3	13.1	<0.001
*Healthy foods time consuming*				
Disagree	57.4	33.8	8.8	
Agree	48.0	37.9	14.1	<0.001
*Healthy foods aren't tasty*				
Disagree	57.0	34.2	8.8	
Agree	48.2	36.6	15.2	<0.001
*Health considerations influence food choice*				
Disagree	41.8	39.0	19.2	
Agree	58.2	33.7	8.1	<0.001

### Multilevel statistics

#### Density

In unadjusted models, density was a significant predictor of both monthly (OR 1.07; 95% CI 1.00 – 1.13) and weekly (OR 1.10; 95% CI 1.02 – 1.18) fast food purchasing (Table 2). After adjustment for age, country of birth and household composition, density was only a significant predictor for weekly fast food purchasing (OR 1.09; 95% CI 1.01 – 1.19). The inclusion of individual socio-economic predictors attenuated this association to non-significance.

**Table 2:** Multilevel multinomial regression models of fast food restaurant access as a predictor of fast food purchasing

	Model One		Model Two		Model Three		Model Four		Model Five	
	Unadjusted		Adjusted for socio-demographic		Model 2 + socio-economic		Model 3 + area-level disadvantage		Model 4 + attitude	
	OR	(95% CI)	OR	(95% CI)	OR	(95% CI)	OR	(95% CI)	OR	(95% CI)
*Density of fast food stores*										
Monthly purchasing	1.07	(1.00 – 1.13)*	1.06	(0.99 – 1.14)	1.05	(0.99 – 1.11)	1.04	(0.98 – 1.11)	1.05	(0.98 – 1.11)
Weekly purchasing	1.10	(1.02 – 1.18)*	1.09	(1.00 – 1.18)*	1.07	(0.99 – 1.15)	1.04	(0.96 – 1.13)	1.05	(0.97 – 1.14)
*Variety of fast food stores*										
Monthly purchasing	1.16	(1.05 – 1.28)**	1.15	(1.04 – 1.28)**	1.13	(1.03 – 1.24)**	1.13	(1.02 – 1.24)*	1.13	(1.02 – 1.25)*
Weekly purchasing	1.21	(1.07 – 1.37)**	1.19	(1.04 – 1.36)*	1.13	(1.00 – 1.29)*	1.10	(0.96 – 1.25)	1.11	(0.97 – 1.27)
*Proximity to the nearest store (km)*										
Monthly purchasing	0.88	(0.74 – 1.05)	0.88	(0.73 – 1.06)	0.89	(0.76 – 1.06)	0.91	(0.77 – 1.07)	0.90	(0.76 – 1.07)
Weekly purchasing	0.77	(0.62 – 0.96)*	0.77	(0.61 – 0.97)*	0.82	(0.65 – 1.02)	0.85	(0.67 – 1.07)	0.82	(0.65 – 1.03)

* p-value significant at < 0.05 level

** p-value significant at <0.01 level

*** p-value significant at <0.001 level

Reference group: never eat fast food

#### Variety

An independent association was found to exist between the variety of fast food restaurants and monthly fast food purchasing. An increase of one different fast food chain within the three kilometre network areas increased the odds of monthly fast food purchasing by 13% in the fully adjusted model (OR 1.13; 95% CI 1.02 – 1.25) (Table 2). In a model unadjusted for confounders, variety was also a significant predictor of weekly fast food purchasing (OR 1.21; 95% CI 1.07 – 1.37). This relationship remained significant after the inclusion of socio-demographic and socio-economic variables but attenuated to non-significance with the inclusion of area-level disadvantage.

#### Proximity

Living closer to the nearest fast food restaurant was a significant predictor of weekly fast food purchasing after adjustment for age, sex and household composition (OR 0.77; 95% 0.61 – 0.97) (Table 2). These effects were attenuated with the inclusion of individual socio-economic predictors. Proximity of fast food restaurants did not predict monthly fast food purchasing.

## Discussion

Results from our study revealed an independent association between the variety of fast food restaurants and fast food purchasing; an increase of one different fast food chain within the 3 kilometre network areas increased the odds of monthly fast food purchasing by 13%. However, out of six relationships tested this was the only significant findings in fully adjusted models. No significant relationships were found between density and proximity after the inclusion of individual socio-economic predictors suggesting these were important confounders.

It is important to note that although we only had one significant finding in models adjusted for all confounders the relationships were all in the same direction; they were all suggestive of a possible relationship between greater access and increased purchasing. It is possible that the lack of significant findings, particularly for those in the weekly purchasing category, were due to a lack of power at both the individual level (only 10% of respondents) and area level (only 49 areas).

Our analysis examined both infrequent and frequent fast food purchasing. This was represented by the categories monthly (one to three times per month) and weekly (four or more times per month) bearing in mind that this represented fast food consumed at home only and is likely to be an underestimate of total fast food consumption. For each access measure the strength of the association was marginally stronger for weekly than monthly fast food purchasing in the unadjusted models. Adjustment for confounding variables tended to attenuate the estimates for weekly fast food purchasing towards the null. After adjusting for confounders, the estimates for density and variety were of similar magnitude for both weekly and monthly purchasing however proximity remained more strongly associated with weekly purchasing. This similarity in the strength of association for monthly and weekly purchasing for density and variety is difficult to explain. Given that the odds for both monthly and weekly purchasing are a comparison of those who never purchase fast food it may simply be that never purchasing is more likely when fast food restaurants are absent and that the presence of fast food restaurant increased the likelihood of purchasing irrespective of whether someone purchases infrequently or frequently.

In all models and for both monthly and weekly purchasing, variety of fast food restaurants produced higher odds than the density measure. In effect variety is a measure of choice rather than a simple count of stores as indicated by the density measure. A density of three stores nearby may be irrelevant if the three stores are from the same chain. However if they are from different chains than nearby residents are provided with the choice of a wider range of fast food products and a greater choice means a higher likelihood that taste preferences of residents can be matched. Although the odds for proximity were relatively strong, this was found to be a non-significant predictor of purchasing. This may be because distance to the nearest store is not overly important given that the purchasing measure is based consumption at home in which case most trips would be undertaken using a vehicle and therefore an extra few hundred metres to the nearest store becomes less of an access barrier.

Previously, US findings showed that the frequency of fast food consumptions was not associated with the number of outlets near respondents home or work address [6]. Multilevel level analysis from within Australia also did not find any association between fast food access and purchasing [7]. We suggested that these null findings may have been linked to a number of potential methodological problems previously discussed including 'aggregation error' [8], lack of specificity between the outcome and exposure variables [7], and poorly conceptualise models that do not account for confounding, particularly in the case of attitude variables [12,13] and taste preferences [9,10]. In addressing these methodological issues we also found that fast food purchasing was not associated with the density and proximity but showed some association with the variety of fast food restaurants in a local area. Therefore despite what appears to be mounting evidence to the contrary, a potential link between access and purchasing cannot be discounted and further research is required.

Other important results include the bivariate analyses. Literature has shown that the variables presented in Table 1 are potential determinants of both purchasing and fast food restaurant locations [1,2,6,7,34-39] and our descriptive results support this in relation to purchasing. However, the relationships presented here are complex and the causal association between these variables are unexplored. This is because these results are presented as proportions which are unadjusted for confounding. A deeper understanding of the association of these variables to fast food purchasing would require a separate conceptual model with each predictor modelled separately adjusting for confounders. This would require further unpacking of our DAG presented in Figure 1. This would allow us to determine confounders for a specific exposure (e.g. household income or area-level disadvantage) thus reducing the potential for bias which potentially exists within research related to dietary behaviours and neighbourhood health effects [30,40,41]. While these results are suggestive, we recommend that future research carefully disentangle the relationships using DAGs, particularly in relation to assessing the potential mediating role of fast food store access for socio-economic associations and intake.

### Strengths and limitations

Several methodological challenges are addressed in this study. First we avoided potential aggregation error as density, variety, and proximity were calculated from participants' household addresses rather than the centroid of their CCD. This is in contrast to Turrell and Giskes [7] used the centroid of the geographical area (CCD) to construct their access measures. Second, participants were asked about the frequency with which they purchased fast food from each of the five fast food restaurants used in the exposure variables. This ensures specificity between the measurements of access and individual fast food purchasing. Previous studies have not collected information on fast food purchasing in this way or have used unrelated measures of exposure to fast food. Third, to address endogeneity our models included a number of potential confounders that might drive the demand for fast food such as individual and area socio-economic characteristics, attitudes and taste preferences. This final point was critical when conceptualising the covariates to include in this analysis as represented in our causal model in Figure 1. The use of a DAG enabled the identification of an independent association between supply (or accessibility) of fast food and purchasing. Researchers have previously argued that the association between fast food access and consumption may be overestimated because variables such as taste preferences are not accounted for [10]. The inclusion of attitude variables and taste preferences into our models is a considerable advance on previous analyses. However the main attenuation of the associations between access and purchasing came through the inclusion of individual socioeconomic variables in the models which suggests that these predictors are more likely to be confounders of potential associations than variables related to attitudes.

In addition to addressing aggregation error, specificity and confounding we improved on previously used measures of access by including a measure of variety as well as density and proximity. We are unaware of other studies which include a measure of variety. The two previous studies [6,7] include a measure of density (although Jeffery *et al*. call this "proximity") while Turrell and Giskes [7] also use a measure of proximity. The variety variable offers something new in that it allows us to measure different choices available to residents rather than the quantity of restaurants or ease of access. Further, although Jeffery *et al*. [6] measured access from individual locations, our study improves on this by using network distance rather than Euclidean distance.

We note that our study also has some limitations. First, the definition of fast food was restricted to five major franchised fast food restaurants. To date no universal definition exists as to what constitutes fast food or a fast food restaurant, a fact often discussed in the public health literature [6,42,43]. The use of these chain brands allowed us to align purchasing and access data. Second, the study only examined fast food purchased for home consumption is likely be an underestimate of the total fast food consumed. However, access to fast food restaurants in local residential environments is likely to impact on purchasing of fast food for consumption at home rather than consumption away from home again improving the specificity of the exposure and outcome. Third, there is potentially a much wider range of variables that capture attitudes to food purchasing choices [12,13] that may also be confounders of the association between fast food restaurant access and purchasing. However, our inclusion of variables on attitudes and taste was a considerable advance on previous analyses. Further, when we did include variables for attitudes and taste, they did not attenuate the effect estimates. Thus, although confounding due to these variables may be a theoretical concern [9,10,12,13], in practice they may not be important confounders. Fourth, although our results are suggestive of an association between access to fast food and fast food purchasing most of the estimates were not statistically significant after controlling for confounding. It is likely that the study was underpowered to detect these area level effects (only 49 small areas) and larger multilevel studies are needed. Finally, we did not account for potential selection effects; it is theoretically possible that people may choose to live in a particular area because of access to fast food restaurants [9]. In addition, the Moving To Opportunity study, the only study that has randomised households to different location (high and low poverty level), has demonstrated beneficial effects of moving from a high poverty to a low poverty neighbourhood on body mass index and mental health. These findings are consistent with observational evidence suggesting that selection effects may be less important than previously argued, at least for these two outcomes [16,44].

### Implications of the study

The results from our study have suggested that a reduction in accessibility to fast food restaurants, particularly variety, may result in a reduction in purchasing leading to a better dietary profile. However we remain cautious about the importance of this finding given that no significant association was reported for other measures of access. Further research is required to examine more thoroughly the associations between unhealthy food environments and unhealthy dietary behaviours.

## Conclusion

We provide some evidence that fast food purchasing is more likely when people have access to a wider variety of fast food restaurants but we did not find evidence of an effect when access to a greater density or closer proximity were examined. There has been a limited amount of research investigating this topic thus far however the work that has been undertaken provides a useful basis for comparison. It is hoped that some of the methodological issues we raised may help guide future research as regards to defining and measuring accessibility and accounting for confounders.

## Competing interests

The authors declare that they have no competing interests.

## Authors' contributions

LT drove the design of this study, conducted the analysis and wrote the first draft of this paper. RB and AK contributed to the study design and the redrafting of the paper. RB undertook the multiple imputation of the data used for analysis.
